# Global prevalence of COVID-19-induced acute respiratory distress syndrome: systematic review and meta-analysis

**DOI:** 10.1186/s13643-023-02377-0

**Published:** 2023-11-13

**Authors:** Abere Woretaw Azagew, Zerko Wako Beko, Yohannes Mulu Ferede, Habtamu Sewunet Mekonnen, Hailemichael Kindie Abate, Chilot Kassa Mekonnen

**Affiliations:** Department of Medical Nursing, School of Nursing, College of Medicine and Health Sciences, University of Gondar, Gondar, Ethiopia

**Keywords:** COVID-19, Acute respiratory distress syndrome, ARDS, Global

## Abstract

**Background:**

Acute respiratory distress syndrome (ARDS) is potentially a fatal form of respiratory failure among COVID-19 patients. Globally, there are inconsistent findings regarding ARDS among COVID-19 patients. Therefore, this study aimed to estimate the pooled prevalence of COVID-19-induced ARDS among COVID-19 patients worldwide.

**Methods:**

To retrieve relevant studies, the authors searched Embase, MEDLINE, PubMed, Web of Science, Cochrane Library, Google, and Google Scholar using a combination of search terms. The search was conducted for articles published from December 2019 to September 2022. Articles were searched and screened by title (ti), abstract (ab), and full-text (ft) by two reviewers independently. The quality of each included article was assessed using the Newcastle–Ottawa Assessment Scale. Data were entered into Microsoft Word and exported to Stata version 14 for analysis. Heterogeneity was detected using the Cochrane *Q* statistics and *I*-square (*I*^2^). Then the sources of variations were identified by subgroup and meta-regression analysis. A random effect meta-analysis model was used. The publication bias was detected using the graphic asymmetry test of the funnel plot and/or Egger’s test (*p* value < 0.05). To treat the potential publication bias, trim and fill analysis were computed. The protocol has been registered in an international database, the Prospective Register of Systematic Reviews (PROSPERO) with reference number: CRD42023438277.

**Results:**

A total of 794 studies worldwide were screened for their eligibility. Of these 11 studies with 2845 participants were included in this systematic review and meta-analysis. The overall pooled prevalence of COVID-19-induced ARDS in the world was found to be 32.2% (95%CI = 27.70–41.73%), *I*^2^ = 97.3%, and *p* value < 0.001).

**Conclusion:**

The pooled prevalence of COVID-19-induced ARDS was found to be high. The virus remains a global burden because its genetic causes are constantly changing or it mutated throughout the pandemic to emerge a new strain of infection. Therefore, interventions such as massive vaccination, early case detection, screening, isolation, and treatment of the cases need to be implemented to tackle its severity.

**Supplementary Information:**

The online version contains supplementary material available at 10.1186/s13643-023-02377-0.

## Background

The coronavirus-2019 (COVID-19) is a communicable respiratory disease caused by new strains of the coronavirus that causes illness in humans [1]. It originated in Wuhan, Hubei province, China in late December 2019 [2], later it rapidly spread across the world and became a global pandemic [3]. The virus is a highly contagious infectious disease caused by severe acute respiratory syndrome coronavirus 2 [4, 5]. As of July 9, 2023, based on the World Health Organization (WHO) weekly report, there were over 767 million confirmed cases and over 6.9 million deaths globally [6]. The pandemic also affects the global economies which lead to the global economic crisis [7]. It results in about 90 trillion USD loss in the global economies [8].

COVID-19 vaccine utilization reduces the severity of infection, hospitalization, and death [9]. Bivalent booster recipients had higher protection against infection and significantly higher protection against death than unvaccinated persons [10], but the outbreak still exists and causes negative health impacts [11]. This is due to the global population COVID-19 vaccine hesitancy [12], absence of definitive medical therapy [13] and prophylaxis [14], changing genetic codes or mutation [15], and the existence of new variants including alpha, beta, gamma, delta, and Omicron [16].

COVID-19 results in multi-organ dysfunctions [17] but predominantly affects the respiratory system causing COVID-19-induced acute respiratory distress syndrome (ARDS) [18]. ARDS is the acute onset of hypoxemia (the ratio of partial pressure of arterial oxygen to fraction of inspired oxygen [PaO2/FiO2] ≤ 200 mmHg), with bilateral infiltrates on frontal chest x-ray, in the absence of left atrial hypertension [19]. It is a fatal complication of COVID-19. The mortality rate of COVID-19 patients with ARDS ranges from 23 to 56% [20]. COVID-19-induced ARDS is the main reason for admission to intensive care units [21]. The treatment of ARDS needs longer hospital stays, advanced medical equipment, and medical therapies, and as a result, the disease increases healthcare costs [22]. The risks of COVID-19-induced ARDS are multifactoral, but it is associated with advanced age, and patients with co-morbid illnesses such as diabetic mellitus, heart disease, chronic obstructive pulmonary disease, and coagulopathy disorder [23–25]. Other risk factors include obesity, smoking, taking immune suppressive medication, and ethnicity [26].

Despite massive vaccination provisions and other preventive strategies, COVID-19 is still a global disease burden and ultimately causes ARDS [27, 28]. Globally, there were inconsistent research findings about the prevalence of COVID-19-induced ARDS [29]. Its prevalence varies across the studies. Therefore, this study aimed to determine the pooled prevalence of COVID-19-induced ARDS in the global population.

### Research question

What is the global prevalence of COVID-19-induced ARDS among COVID-19 patients?

## Methods

### Study protocol registration and reporting

The study protocol has been registered in PROSPERO, an international register of systematic reviews (CRD42023438277). The procedure for this systematic review and meta-analysis was designed following the preferred items for systematic review and meta-analyses (PRISMA-2020) reporting guideline [30].

### Search strategies

The authors searched for articles in Embase, MEDLINE, PubMed, Web of Science, Cochrane Library, Google, and Google Scholar using a combination of search terms. Endnote Version 7 reference management software was used to export, download, organize, review, de-duplicate, and cite the articles. A detailed search was employed using the synonyms of medical subject heading MeSH) terms. Boolean logic operators OR and AND were used to combine search terms. Then the search string expressed as “acute respiratory distress syndrome” OR ARDS OR “adult respiratory distress syndrome” OR “shock lung” AND “COVID-19″ OR “2019 Novel Coronavirus disease” OR “2019 Novel Coronavirus infection” OR”2019-nCoV disease” OR 2019-nCoV infection” OR “COVID-19 pandemic*” OR “COVID-19 virus disease” OR “COVID-19 virus infection” OR “Coronavirus disease 2019″ OR “Coronavirus disease-19″ OR “severe acute respiratory syndrome Coronavirus 2 infection” OR “SARS Coronavirus 2 infection” OR “SARS-CoV-2 infection” AND Worldwide. The search strategy was peer-reviewed. Two authors (CKM & HMK) were searched independently. Articles published from December 2019 to September 2022 were included in the study (Additional file 1).

### Eligibility criteria

Studies were included in the review if they reported on (1) adults (age ≥ 18 years) with COVID-19 patients, (2) observational study designs such as cross-sectional or cohort study designs, (3) articles published in English, and (4) studies from across the world. Whereas, conference papers, qualitative studies, articles with no full text, and published in languages other than English were excluded from the study.

### Screening of the review

Regarding the screening of articles, two reviewers (CHK and HKA) screened the articles independently by title, abstract, and full text. The disagreements between the reviewers were resolved by discussion. Any ongoing disagreements or uncertainty were resolved by involving a third reviewer (YMF).

### Definition of the outcome

ARDS has been diagnosed both clinically and in a radiological investigation by a physician from the patient on admission. Clinically, ARDS is diagnosed when the patient has two or more clinical manifestations such as cough, fever, sore throat, and shortness of breath [31]. Radiologically, ARDS is defined according to Kigali modification which is the presence of bilateral opacities at chest radiograph or lung ultrasound and hypoxia with a cut-off point SPO2/FIO2 less than or equal to 315 [32].

### Quality appraisal

Articles were assessed for their quality using the Newcastle Ottawa assessment scale adapted from the cross-sectional and cohort studies quality assessment tool with a score of 6 out of 10 considered high-quality scores [33, 34]. Two authors (ZWB and HSM) assessed the quality of each paper. The reviewers compared the quality of appraisal scores and resolved inconsistencies before calculating the final appraisal score (Additional file 2). All the included studies had high-quality scores. The PRISMA guideline 2020 [30] was used to report the results of the study.

### Data extraction

The data were extracted by data extraction format using Microsoft Word. The format was prepared and piloted for its aim, relevance, clarity, consistency, and depth of the contents prior to the data extraction. Then all important parameters were extracted from each included study by two reviewers (CKM and HKA) independently. The discrepancies between the two reviewers were managed through discussion and/or involving a third reviewer (YMF). The information such as author(s) of the study, study year/year of publication, study design, sample size, prevalence/incidence of ARDS, data collection technique, and funding sources were extracted (Additional file 3).

### Data analysis

The data were exported into Stata version 14 Software for analysis. Significant heterogeneity was checked by Cochrane Q statistics and/or *I* squared (*I*^2^). The *I*^2^ heterogeneity test statistics results of 20%, 50%, and 75% were declared as low, moderate, and considerably high heterogeneity [35] respectively. The summary effect estimate of the prevalence of ARDS was obtained by using meta-analysis with a random effect model due to high heterogeneity. Subgroup analysis was computed to see the variation across studies using shared study characteristics such as study design, publication year, region of the study, and income. Furthermore, bivariate and multivariate meta-regression analyses were used to identify the covariate that causes the variation. Additionally, sensitivity analysis was also computed to determine the influence of a single study on the pooled estimates. Moreover, the graphic asymmetric of the funnel plot and/or Egger’s test (*p* value < 0.005) were used to declare the presence of publication bias/small study effect [36] and handled by trim and fill analysis using a random effect model.

## Results

### Study selection and characteristics of the studies

**Fig. 1:**
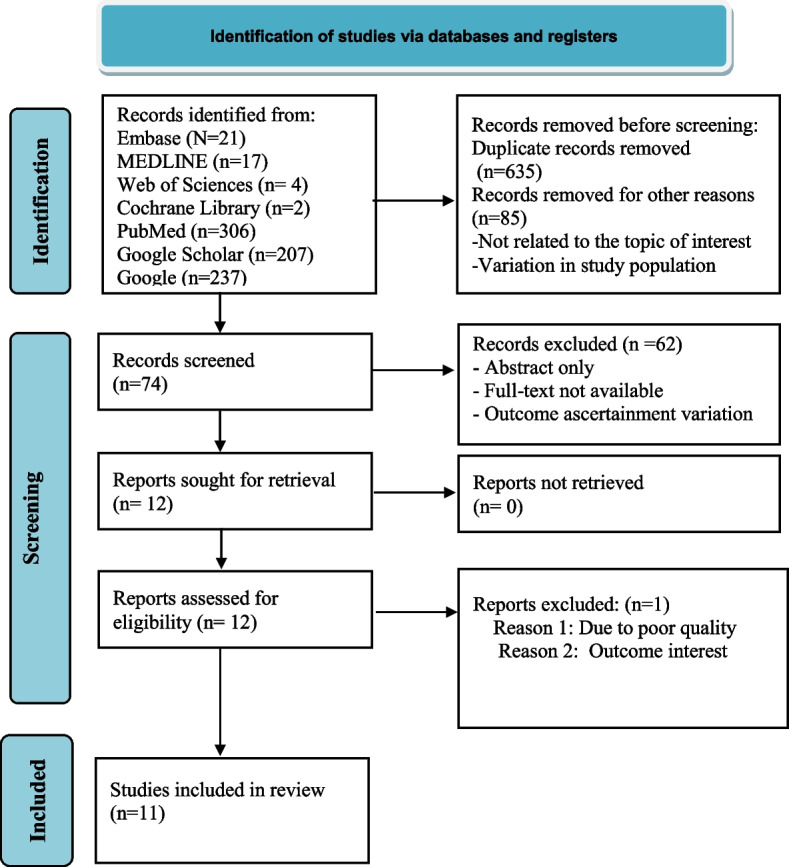
PRISMA flow diagram for the flow of information through the phases of the review

The search strategy retrieved 794 original research articles. After the removal of the duplicates, articles unrelated to the topic of interest, and variation in the study population seventy-four articles remained. Following further screening, sixty-two articles were removed because of abstract only, full-text not available, and variation in the outcome ascertainments. The authors made an effort to gate the full-text requests via personal emails of the authors. About twelve full-text articles were accessed for eligibility of which one article was excluded because of reporting without the outcome of interest (Fig. 1). Finally, eleven articles were retrieved and included with a total sample size of 2845 populations. Of these studies, three of them were in China [37–39], three in Ethiopia [21, 40, 41], two in the USA [42, 43], One in Pakistan [44], one in South Africa [45], and one in South Sudan [46]. The prevalence of COVID-19-induced ARDS ranges from 9 [40] to 67.3% [39]. Regarding the publication year of the studies, articles were published between the years 2020 to 2022. On average all the articles had high-quality assessment scores with rating scores ≥ 7 (Table 1).

**Table 1:** Comprise the basic characteristics of the studies and study participants

Authors/year	Country	Study design	Sample size	Data collection technique	Prevalence (%)	Funding source	Quality appraisal score
Getachew H, et al. 2021 [40]	Ethiopia	Cross-sectional	504	Chart review	9	Not funded	6.5
Tolossa, et al. 2022 [21]	Ethiopia	Cross-sectional	318	Chart review	32	Not funded	7.5
Marta, et al. 2021 [45]	South Africa	Cohort	396	Chart review	34.6	European and developing countries	7.5
Kristen, et al. 2021 [46]	South Sudan	Cross-sectional	435	Interviewer administered	38.3	Not reported	7
Chaomin, et al. 2020 [37]	China	Cross-sectional	201	Chart review	41.8	Shanghai Science and Technology Committee	7.5
Suleyman, et al. 2020 [42]	USA	Cross-sectional	463	RRecord review	24	Not reported	7.0
Wang D, et al. 2020 [38]	China	Cross-sectional	138	Record review	15	Not reported	7.0
Yang X et al. 2020 [39]	China	Cross-sectional	52	Record review	67.3	Not funded	6.5
Ayaz A et al. 2020 [44]	Pakistan	Cohort	66	Record review	15	Not reported	7.5
Sultan M, et al. 2021 [41]	Ethiopia	Cross-sectional	92	Record review	25	Not funded	8
Rachel L, et al. 2020 [43]	USA	Cohort	180	Record review	56.7	Not funded	7.5

*USA* United States of America

### The global prevalence of COVID-19-induced ARDS

**Fig. 2:**
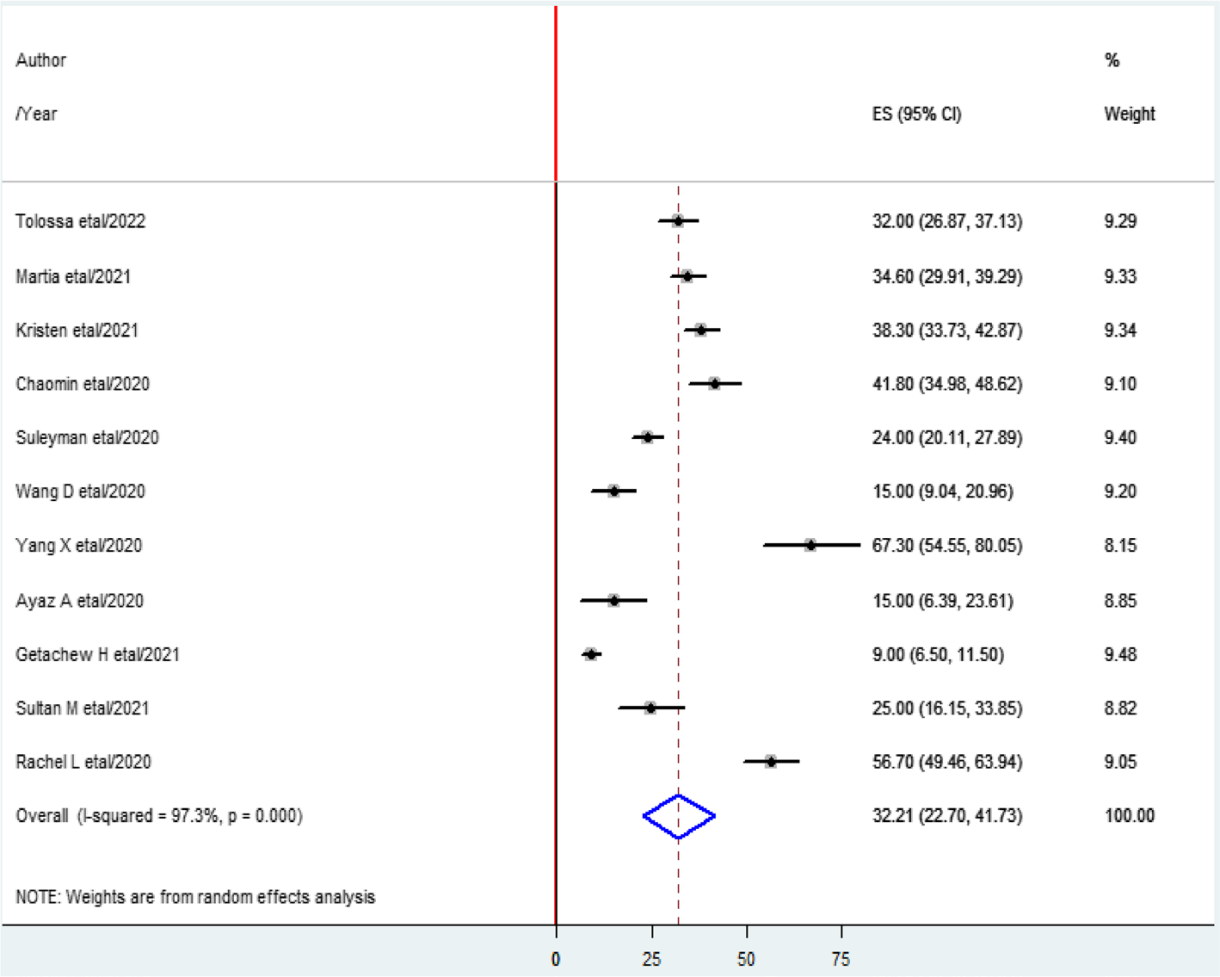
Forest plot shows the pooled prevalence of acute respiratory distress syndrome

The overall pooled prevalence of COVID-19-induced ARDS was found to be 32.2% (95%CI 27.7–41.73%), *I*^2^ = 97.3%, and *p* value < 0.001 (Fig. 2).

### Heterogeneity test

As shown in the Fig. 2 above, the heterogeneity test (*I*^2^) of the study was 97.3% with a *p* value < 0.001. This indicates there is considerable variability across the studies. Then subgroup and Meta-regression analyses were computed to identify the cause/source of the variations.

### Subgroup analysis

The source of heterogeneity was further assessed using the study design (cohort and cross-sectional, publication year (≥ 2021 and < 2021), region of the study (Asia, Africa, and USA), and income status (high-income countries (HICs)) and Low-and middle-income countries (LMICs)). As it is shown in the table; there were still high variations across study characteristics such as study design, publication year, and region of the studies (Table 2).

**Table 2:** Subgroup analysis on COVID-19-induced ARDS

Subgroups	Criteria	Number of studies	Prevalence	Heterogeneity statistics	*p* value	*I*^2^	Tau squared
Study design	Cros-sectional	8	30.91 (20.27–41.54)	254.57	*P* < 0.001	97.3%	223.5
	Cohort	3	35.54 (15.32–55.77)	54.57	*P* < 0.001	96.3%	306.5
Publication year	≥ 2021	5	27.75 (13.75–41.75)	194.54	*P* < 0.001	97.9%	243.3
	< 2021	6	36.17 (21.68–50.65)	141.76	*P* < 0.001	96.5%	311.5
Region of the study	Africa	5	27.75 (13.75–41.75)	194.54	*P* < 0.001	97.9	247.3
	Asia	4	34.28 (13.86–54.69)	79.27	*P* < 0.001	96.2	413.5
	USA	2	40.20 (8.16–72.25)	60.83	*P* < 0.001	98.4	525.9
Income status	HICs	5	40.39 (24.04–56.73)	129.31	*P* < 0.001	96.9	331.9
	LMICs	6	25.7 (13.35–38.05)	196.32	*P* < 0.001	97.5	228.6

*HICs* High-income Countries, *LMICs* Low-income countries, *USA* United States of America

### Meta-regression

Furthermore, a univariate meta-regression was carried out with sample size and publication year for possible heterogeneity. The result of the analysis indicates that sample size and publication years are not the sources of variation across the studies (Table 3).

**Table 3:** Meta-regression analysis of studies on COVID-19-induced ARDS

Heterogeneity	Coefficient	Std. Err	*P* value	95% CI
Sample size	− 0.002	0.0061433	0.756	− 0.016–1.012
Publication year	− 0.14	1.85714	0.941	− 4.424–4.14

### Publication bias of the studies

**Fig. 3:**
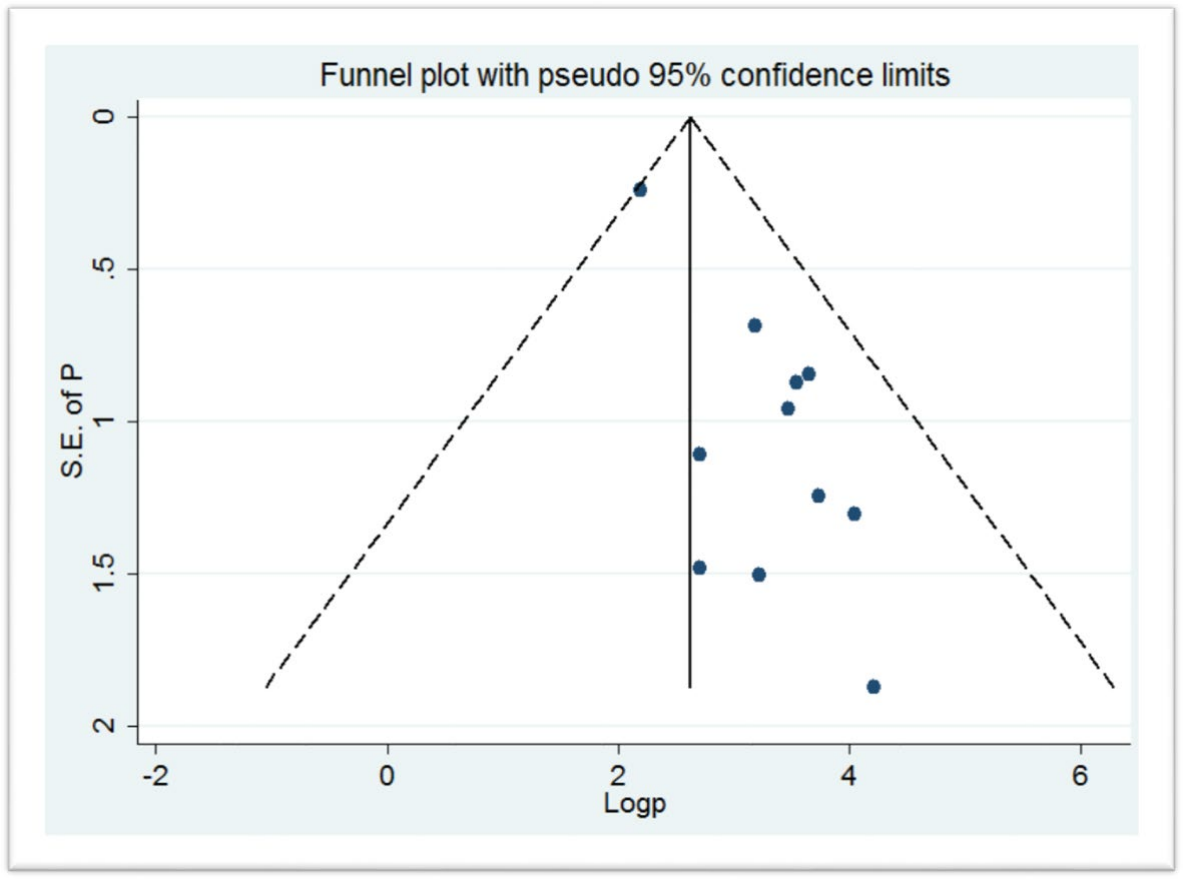
Funnel plot shows the publication bias of the included studies

The publication bias was evaluated using the funnel plot asymmetry and Egger’s test (*p* value < 0.05). The funnel plot showed asymmetrical distribution (Fig. 3), and Egger’s test *p* value is < 0.001, which is significant meaning there is a publication bias.

### Sensitivity analysis

**Fig. 4:**
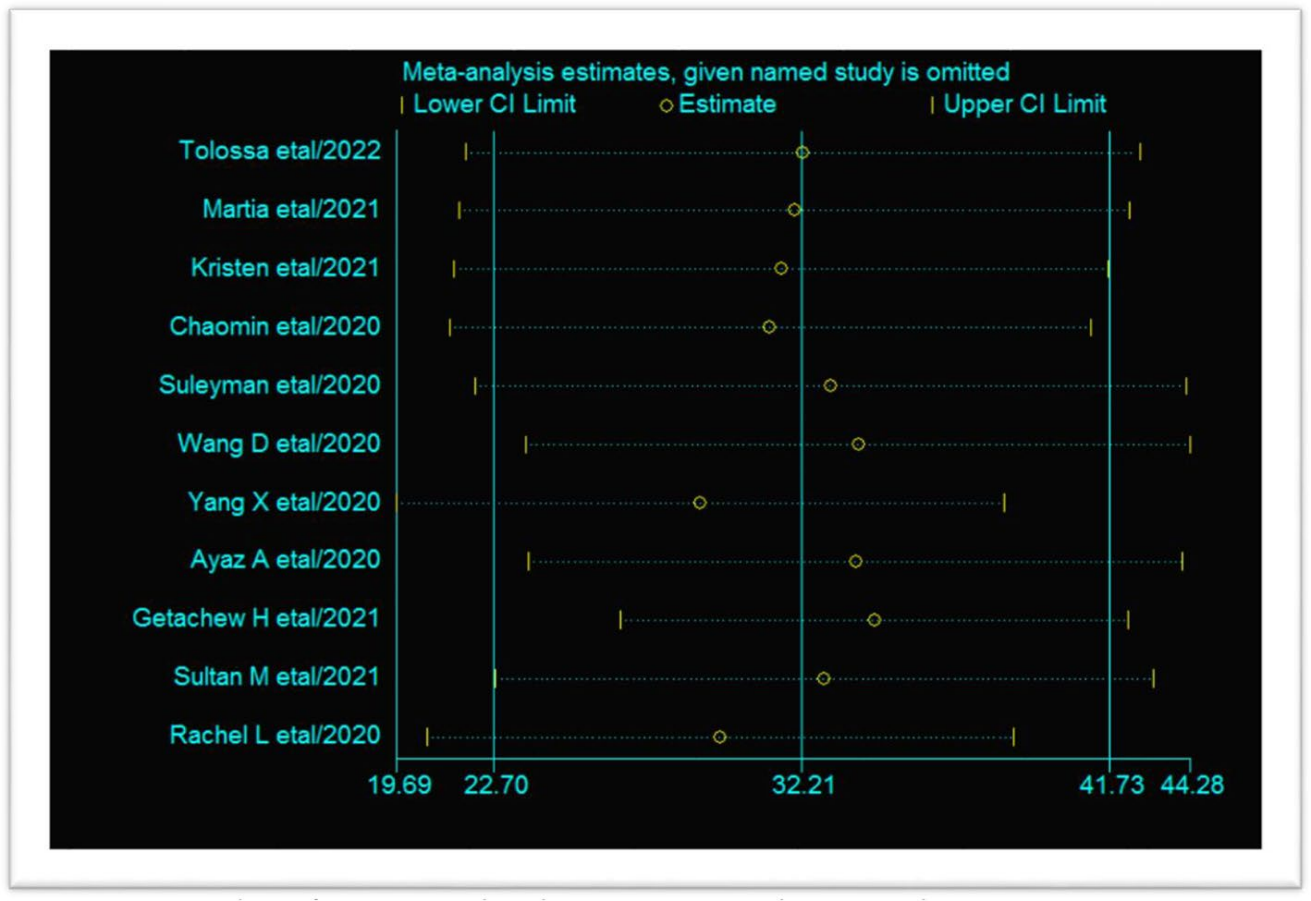
Sensitivity analysis of COVID-19-induced acute respiratory distress syndrome

The effect of a single study on the pooled estimate was detected. The findings showed that the point estimate of each study was within the lower and upper limits. This indicates there is no influence of a single study on the pooled estimates (Fig. 4).

### Trim and fill analysis

**Fig. 5:**
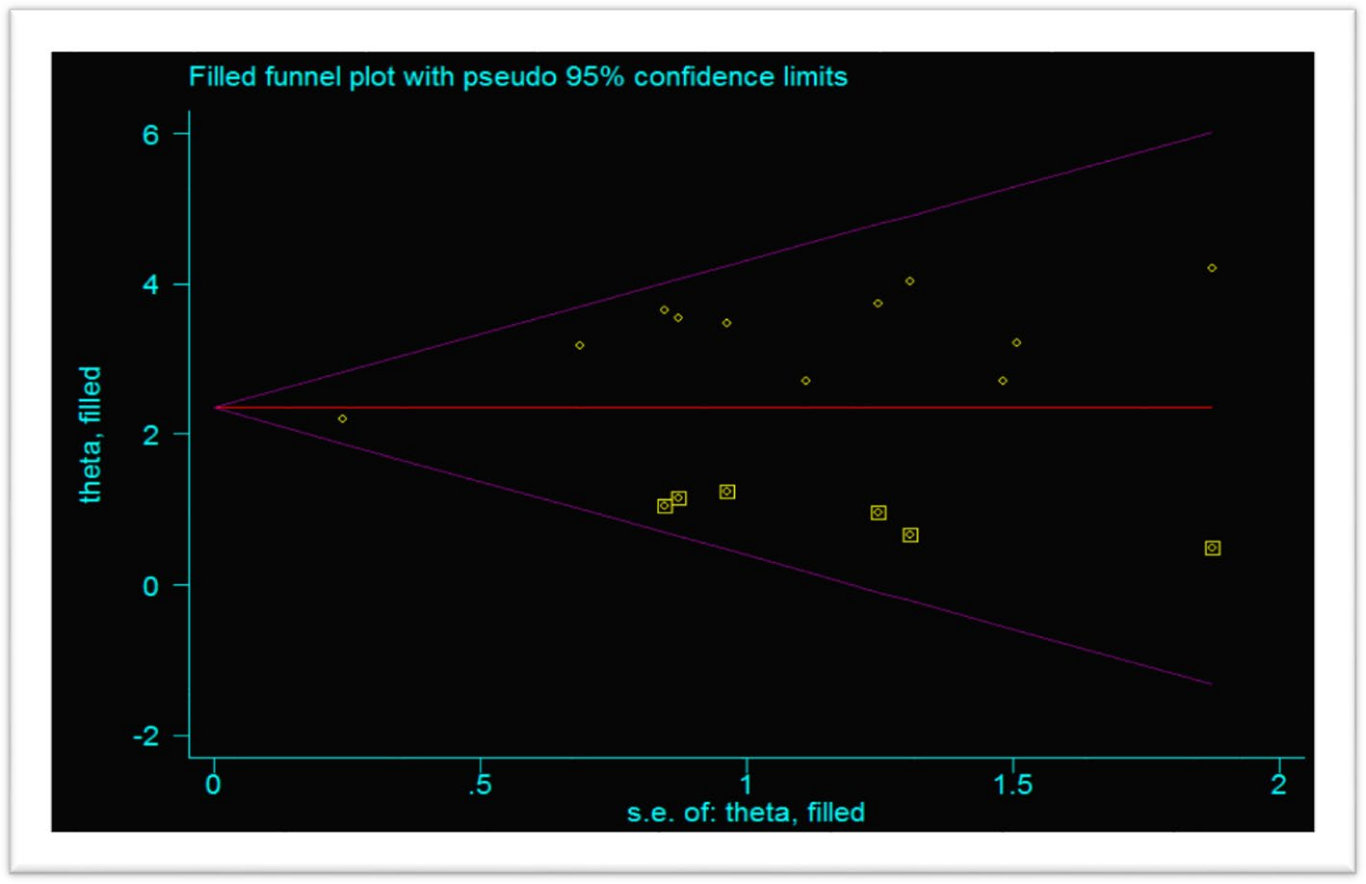
Trim and fill analysis of COVID-19-induced acute respiratory distress syndrome

This systematic review showed a considerable publication bias, so a non-parametric trim and fill analysis was conducted. After a number of iterations/cycles, six articles were included making a total of seventeen studies (Fig. 5).

## Discussion

This systematic review and meta-analysis revealed that the pooled prevalence of COVID-19-induced ARDS worldwide was found 32.2% (95% CI = 27.70–41.73%. The finding was higher than the large-scale observational cohort study conducted in Pennsylvania 6.2% [47], China (24%) [48], and the USA (19%) [49]. The discrepancy might be due to differences in the case definitions, population characteristics, and study design. The finding is also much higher than the national representative large data study in patients hospitalized with COVID-19 in Poland with a prevalence of 3.6% [50]. The discrepancy might be the current systematic review included Up To Date studies. This implied that as time increased, there may be increasing COVID-19-related complications. The high pooled prevalence of COVID-19-induced ARDS indicated that COVID-19 significantly impacts the overall health of the patients. COVID-19-associated ARDS includes severe pulmonary infiltration, edema, and inflammation, leading to impaired alveolar homeostasis and alteration of pulmonary physiology [51].

ARDS severity is characterized by inadequate tissue oxygenation and the occurrence of non-compliant lungs [52]. ARDS and COVID-19 share a close relationship as both are predators of the respiratory system. As studies revealed the median time from symptoms onset to ARDS is longer among COVID-19-induced ARDS compared to classical ARDS [53].

The heterogeneity test (*I*^2^) of the current study was 97.3% with a *p* value < 0.001. The finding indicates there is considerable variability across the studies. This is due to the variation in population characteristics such as the presence of co-morbidities, lifestyle of the population (smoking and obesity), and socio-economic status [54]. Furthermore, the variation may be attributed to study characteristics including outcome measurement and design of the study [55].

This systematic review and meta-analysis have their own strengths. The study can provide evidence for the global community to act on the problem. The authors used a more comprehensive assessment and screening method to include and exclude studies that were published globally. On the contrary, the study has some important limitations. The study did not include patients under 18 years old because the COVID-19 severity is higher in the adult population. The authors used articles published in English which may miss articles published in other languages resulting in an over/under estimate of the prevalence of COVID-19-induced ARDS. Furthermore, the study did not include the patient treatment outcomes and factors related to COVID-19-induced ARDS. As a result, the authors recommended further research to be conducted on patient treatment outcomes and contributing factors to enhance the clinical outcome of patients.

## The implication of the study

The study highlights the global prevalence of COVID-19-induced ARDS among adults. The study provides an opportunity to give attention to reducing COVID-19-induced ARDS morbidity and mortalities through early screening and providing evidence-based interventions.

## Conclusion

The pooled prevalence of ARDS among COVID-19 patients was found to be high, which needs an effort to combat its morbidity and mortality. The virus remains a global burden its genetic causes are constantly changing or it mutating throughout the pandemic to emerge a new strain of infection. Therefore, interventions such as massive vaccination, early case detection, screening, isolation, and treatment of the cases need to be implemented to tackle its progression and severity.

### Supplementary Information

**Additional file 1.** Search string.

**Additional file 2.** Newcastle-Ottawa quality assessment checklist.

**Additional file 3.** Data extraction.

## Data Availability

The data supporting the findings of this study is available from the corresponding author and the author will share as per the request of the reviewer.

## Abbreviations

ARDS: Acute respiratory distress syndrome
COVID-19: Coronavirus diseases 2019
PRISMA: Preferred Reporting Item of Systematic Review and Meta-Analysis
USA: United States of America
WHO: World Health Organization
