# Supplementary material for: Global prevalence of COVID-19-induced acute respiratory distress syndrome: systematic review and meta-analysis
Source: Syst Rev. 2023 Nov 13;12:212. doi: 10.1186/s13643-023-02377-0 (PMC10644454; doi:10.1186/s13643-023-02377-0)
Supplement: Supplementary file 1 — Additional file 1. Search string. [file 13643_2023_2377_MOESM1_ESM.docx]

| **MeSH Headings** | **Entry terms** | **Combinations/search string** | **Searching filters** |
| --- | --- | --- | --- |
| Respiratory distress syndrome | - Acute Respiratory Distress Syndrome - Adult Respiratory Distress Syndrome - Shock Lung | ((((("Respiratory distress syndrome"[Title/Abstract]) OR ("Acute Respiratory Distress Syndrome"[Title/Abstract])) OR (ARDS[Title/Abstract])) OR ("Adult Respiratory Distress Syndrome"[Title/Abstract])) OR ("shock lung"[Title/Abstract]) AND ((ffrft[Filter]) AND (fha[Filter]) AND (observational study[Filter]) AND (fft[Filter]))) AND ((((((((((((("COVID 19"[Title/Abstract]) OR ("2019 Novel Coronavirus Disease"[Title/Abstract])) OR ("2019 Novel Coronavirus Infection"[Title/Abstract])) OR ("2019-nCoV Disease"[Title/Abstract])) OR ("2019-nCoV Infection"[Title/Abstract])) OR ("COVID-19 Pandemic*"[Title/Abstract])) OR ("COVID-19 Virus Disease"[Title/Abstract])) OR ("COVID-19 Virus Infection"[Title/Abstract])) OR ("Coronavirus Disease 2019"[Title/Abstract])) OR ("Coronavirus Disease-19"[Title/Abstract])) OR ("SARS Coronavirus 2 Infection"[Title/Abstract])) OR ("SARS-CoV-2 Infection"[Title/Abstract])) OR ("Severe Acute Respiratory Syndrome Coronavirus 2 Infection"[Title/Abstract])) | **Study design**  (observational study)  **year of publication**  B/n 2020 & 2023 |
| COVID 19 | - 2019 Novel Coronavirus Disease - 2019 Novel Coronavirus Infection - 2019-nCoV Disease - 2019-nCoV Infection - COVID-19 Pandemic - COVID-19 Virus Disease - COVID-19 Virus Infection - Coronavirus Disease 2019 - Coronavirus Disease-19 - Severe Acute Respiratory Syndrome Coronavirus 2 Infection - SARS Coronavirus 2 Infection - SARS-CoV-2 Infection |  |  |
