# Supplementary material for: Global prevalence of COVID-19-induced acute respiratory distress syndrome: systematic review and meta-analysis
Source: Syst Rev. 2023 Nov 13;12:212. doi: 10.1186/s13643-023-02377-0 (PMC10644454; doi:10.1186/s13643-023-02377-0)
Supplement: Supplementary file 2 — Additional file 2. Newcastle-Ottawa quality assessment checklist. [file 13643_2023_2377_MOESM2_ESM.docx]

|  | Getachew H, etal/2021 | Tolossa,  etal/2022 | Kristen,  etal/2021 | Chaomin,  etal/2020 | Suleyman,  etal/2020 | Wang D, etat/2020 | Yang X etal/2020 | Sultan M, etal/2021 |
| --- | --- | --- | --- | --- | --- | --- | --- | --- |
| Selection  1 .Representativeness of the sample:  Truly representative of the average in the target population. |  |  |  |  |  |  |  |  |
| - 1. Somewhat representative of the average in the target group. * | * | * | * | * | * | * | * | * |
| - 1. Selected group of users/convenience sample. |  |  |  |  |  |  |  |  |
| - 1. No description of the derivation of the included subjects |  |  |  |  |  |  |  |  |
| 1. Sample size:    1. Justified and satisfactory (including sample size calculation). * | * | * | * | * | * | * | * | * |
| - 1. Not justified. |  |  |  |  |  |  |  |  |
| - 1. No information provided |  |  |  |  |  |  |  |  |
| 1. Non-respondents:    1. Proportion of target sample recruited attains pre-specified target or basic summary of non-respondent characteristics in sampling frame recorded. * | * | * | * | * | * | * | * | * |
| - 1. Unsatisfactory recruitment rate, no summary data on non-respondents. |  |  |  |  |  |  |  |  |
| - 1. No information provided |  |  |  |  |  |  |  |  |
| 1. Ascertainment of the exposure (risk factor):    1. Vaccine records/vaccine registry/clinic registers/hospital records only. ** |  | * |  |  | * |  | * |  |
| - 1. Parental or personal recall and vaccine/hospital records.* | * |  | * | * |  | * |  | * |
| - 1. Parental/personal recall only. |  |  |  |  |  |  |  |  |
| 1. **Comparability**:   Comparability of subjects in different outcome groups on the basis of design or analysis. Confounding factors controlled.   - 1. Data/ results adjusted for relevant predictors/risk factors/confounders e.g. age, sex, time since vaccination, etc. ** | * | * | * | * | * | * | * | * |
| - 1. Data/results not adjusted for all relevant confounders/risk factors/information not provided. |  |  |  |  |  |  |  |  |
| 1. **Outcome ascertainment** 2. **Assessment of outcome**    1. Independent blind assessment using objective validated laboratory methods.** |  |  |  |  |  |  |  |  |
| 1. Unblinded assessment using objective validated laboratory methods.** |  |  |  |  |  |  |  |  |
| 1. Used non-standard or non-validated laboratory methods with gold standard. * |  |  |  |  |  |  |  |  |
| - 1. No description/non-standard laboratory methods used. | * | * | * | * | * | * | * | * |
| 1. **Statistical test:**    1. Statistical test used to analyse the data clearly described, appropriate and measures of association presented including confidence intervals and probability level (p value). | * | * | * | * | * | * | * | * |
| - 1. Statistical test not appropriate, not described or incomplete. |  |  |  |  |  |  |  |  |
