# Supplementary material for: Global prevalence of COVID-19-induced acute respiratory distress syndrome: systematic review and meta-analysis
Source: Syst Rev. 2023 Nov 13;12:212. doi: 10.1186/s13643-023-02377-0 (PMC10644454; doi:10.1186/s13643-023-02377-0)
Supplement: Supplementary file 3 — Additional file 3. Data extraction. [file 13643_2023_2377_MOESM3_ESM.docx]

**Data abstraction**

| **S. no** | **Author (s)** | **year of public**  **cation** | **country** | **Study**  **popu**  **lation** | **study**  **design** | **Sample**  **size** | **Data collection**  **technique** | **Preva**  **lence**  **of Covid-19 IARDS (P)** | **Sep** | **logp** | **Log**  **sep** | **log**  **odds** | **Selog**  **odds** | **Funding**  **source** | **Quality appraisal score** |
| --- | --- | --- | --- | --- | --- | --- | --- | --- | --- | --- | --- | --- | --- | --- | --- |
| 1 | Getachew H, etal | 2021 | Ethiopia | COVID-19 patients | Crosssectional | 504 | chart  review | 9 | 2.615868 | 3.465736 | 0.961596 | -0.75377 | 0.12014 | Not funded | 6.5 |
| 2 | Tolossa, etal | 2022 | Ethiopia | COVID-19 patients | Crossectional | 318 | chart  review | 32 | 2.390448 | 3.543854 | 0.871481 | -0.63667 | 0.105642 | Not funded | 7.5 |
| 3 | Marta, etal | 2021 | South Africa | COVID-19 patients | Cohort | 396 | chart  review | 34.6 | 2.330759 | 3.64545 | 0.846194 | -0.47683 | 0.098587 | European &  Developing Countries | 7.5 |
| 4 | Kristen, etal | 2021 | South Sudan | COVID-19 patients | Cross sectional | 435 | interviewer administered | 38.3 | 3.478977 | 3.732896 | 1.246738 | -0.33099 | 0.14301 | Not reported | 7 |
| 5 | Chaomin, etal | 2020 | China | COVID-19 patients | Crosssectional | 201 | chart  review | 41.8 | 1.984824 | 3.178054 | 0.68553 | -1.15268 | 0.108857 | Shanghai Science and Technology | 7.5 |
| 6 | Suleyman, etal | 2020 | USA | COVID-19 patients | Crosssectional | 463 | record review | 24 | 3.039594 | 2.70805 | 1.111724 | -1.7346 | 0.241815 | Not reported | 7.0 |
| 7 | Wang D, etat | 2020 | China | COVID-19 patients | Crosssectional | 138 | record review | 15 | 6.505486 | 4.20916 | 1.872646 | 0.721785 | 0.295626 | Not reported | 7.0 |
| 8 | Yang X etal | 2020 | China | COVID-19 patients | Crosssectional | 52 | record review | 67.3 | 4.395245 | 2.70805 | 1.480523 | -1.7346 | 0.261116 | Not funded | 6.5 |
| 9 | Ayaz A etal | 2020 | Pakistan | COVID-19 patients | Cohort | 66 | record review | 15 | 1.274755 | 2.197225 | 0.242754 | -2.31363 | 0.154669 | Not reported | 7.5 |
| 10 | Sultan M, etal | 2021 | Ethiopia | COVID-19 patients | Crosssectional | 92 | record review | 25 | 4.514469 | 3.218876 | 1.507288 | -1.09861 | 0.240772 | Not funded | 8 |
| 11 | Rachel L, etal | 2020 | USA | COVID-19 patients | Cohort | 180 | record review | 56.7 | 3.693169 | 4.037774 | 1.306485 | 0.269622 | 0.150414 | Not funded | 7.5 |

**Notes**: **COVID-19 IARDS**: COVID-19 induced acute respiratory distress syndrome; **Sep**: standard error of prevalence; **logp**: log of prevalence; **Selogodds**: standard error of log odds.
